# Expression of the zinc-finger transcription factor Snail in adrenocortical carcinoma is associated with decreased survival

**DOI:** 10.1038/sj.bjc.6604755

**Published:** 2008-11-18

**Authors:** J Waldmann, G Feldmann, E P Slater, P Langer, M Buchholz, A Ramaswamy, W Saeger, M Rothmund, V Fendrich

**Affiliations:** 1 Department of Surgery, Philipps-Universität Marburg, Marburg, Germany; 2 Department of Pathology and Surgery, Johns Hopkins University School of Medicine, Baltimore, MD, USA; 3 The Sol Goldman Pancreatic Cancer Research Center, Johns Hopkins University School of Medicine, Baltimore, MD, USA; 4 Division of Gastroenterology, Department of Internal Medicine, Philipps-Universität Marburg, Marburg, Germany; 5 Department of Pathology, Philipps-Universität Marburg, Marburg, Germany; 6 Department of Pathology, Marienkrannkenhaus, Hamburg, Germany

**Keywords:** adrenocortical carcinomas, Snail, survival

## Abstract

In this study, we evaluate whether Snail is expressed in adrenocortical cancer (ACC) and if its expression is related to patient outcome. One of the best known functions of the zinc-finger transcription factor Snail is to induce epithelial-to-mesenchymal transition (EMT). Increasing evidence suggests that EMT plays a pivotal role in tumour progression and metastatic spread. Snail and E-cadherin expression were assessed by immunohistochemistry in 26 resected ACCs and real-time quantitative RT–PCR expression analysis was performed. Data were correlated with clinical outcome and in particular with overall patient survival. Seventeen of 26 (65%) ACC tumour samples expressed Snail when assessed by immunohistochemistry. Snail expression was neither detected in normal adrenocortical tissue, nor in benign adrenocortical adenomas. Expression levels were confirmed on the mRNA level by Real-Time–PCR. Survival rates were significantly decreased in Snail-positive tumours compared to Snail-negative tumours: 10 out of 16 *vs* one out of eight patients succumbed to disease after a median follow up of 14.5 and 28.5 months, respectively (*P*=0.03). Patients with Snail-expressing ACCs presented in advanced disease (11 out of 12 *vs* 6 out of 14, *P*=0.01) and tend to develop distant metastases more frequently than patients with negative staining (7 out of 11 *vs* two out of eight, *P*=0.19). In conclusion, we describe for the first time that Snail is expressed in a large subset of ACCs. Furthermore, Snail expression is associated with decreased survival, advanced disease and higher risk of developing distant metastases.

Adrenocortical carcinomas (ACCs) derive from the adrenal cortex. The incidence is about 2 per 1 000 000 per year and ACCs are responsible for 0.2% of cancer-related deaths (Wajchenberg *et al*, 2000). Incidentalomas have been more commonly detected in recent years, possibly because of the availability and use of better imaging techniques. Tumours of more than 4 cm in diameter carry an ∼24% risk of being an ACC (Angeli *et al*, 1997). The clinical course and outcome of patients with ACC vary considerably, with reported overall 5-year survival rates between 38 and 60% (Icard *et al*, 2001; Vassilopoulou-Sellin and Schultz, 2001). A recent study in patients with ACC, which analysed the prognostic factors, revealed the number of distant metastases, number of invaded organs and a high mitotic index as predictors of shorter overall survival (Assie *et al*, 2007).

The oncogenesis of ACC is poorly understood. Germline mutations of the tumour supressor p53 and overexpression of insulin-like growth factor 1 are well known to promote the development of ACC (Edgren *et al*, 1997; Ignaszak-Szczepaniak *et al*, 2006; Slater *et al*, 2006).

In epithelial-to-mesenchymal transition (EMT) epithelial cells show reduced intercellular adhesions and increased mobility, which is normally restricted to fibroblasts or progenitor cells during embryonic development (Zhou and Hung, 2005). Numerous studies support the fact that EMT plays a major role in tumour progression (Zhou and Hung, 2005). During tumour progression, epithelial cells acquire a gene-expression pattern closely resembling that of mesenchymal cells. In consequence cell motility and proteolytic potential increase, possibly facilitating invasive growth and thus contributing to the development of metastases (Thiery and Sleeman, 2006). A hallmark for EMT is the loss of the cell adhesion molecule E-cadherin. Downregulation of E-cadherin can arise – among other mechanisms – through transcriptional repression. Several EMT regulators have been identified as E-cadherin repressors, including the zinc-finger transcription factors Snail (Cano *et al*, 2000), Slug (Hajra *et al*, 2002) and Sip1 (smad-interacting protein 1) (Comijn *et al*, 2001). Snail is degraded through the ubiquitin–proteasome pathway if phosphorylated by glycogen synthase kinase-3*β* (GSK-3*β*). Vice versa inhibition of GSK-3*β* leads to increased expression level of Snail, to a loss of E-cadherin and finally to EMT. Loss of E-cadherin is observed in numerous epithelial tumours and is associated with poorer prognosis (Perl *et al*, 1998). In a transgenic mouse model of breast cancer, Snail expression was associated with a more aggressive phenotype, higher risk of tumour recurrence, and with poor survival rates (Moody *et al*, 2005). Snail expression can be induced by multiple mechanisms, including stimulation by certain cytokines, for example, epidermal growth factor, fibroblast growth factor, hepatocyte growth factor, transforming growth factor-*β* or as downstream target of the wnt-pathway (Cano *et al*, 2000; Huber *et al*, 2005). These growth factors and transcriptional regulators are upregulated in ACC which, was observed in microarray analyses (Giordano *et al*, 2003; Tissier *et al*, 2005; Slater *et al*, 2006) and might support the impact of EMT in this tumour entity.

Recently, our group described that Snail is overexpressed in a large subset of neuroendocrine tumours of the ileum, presenting the first evidence of Snail expression in endocrine tumours (Fendrich *et al*, 2007).

The aim of this study was to assess if Snail is expressed in ACC and if this expression is correlated with clinical outcome and in particular, with overall survival.

## Patients and methods

### Patients and pathology

A series of 26 primary ACCs, one liver metastasis and two lymph node metastases (LNM) of ACCs, were obtained from the tissue bank of the Department of Pathology, Philipps University of Marburg, Germany. Histological diagnosis was confirmed by two experienced pathologists (AR, WS). Histopathological diagnosis and grading were done in accordance to Weiss, van Slooten and Hough (Hough *et al*, 1979; van Slooten *et al*, 1985; Weiss *et al*, 1989). Two patients did not reach a score, which indicate malignancy (P6 and P26), but revealed high Ki67-indexes of 10 and 30%. In addition, they presented with large tumours (130 and 70 mm) and showed aldosteron or cortisol and testosteron excess, respectively. The immunohistochemistry results for Snail were scored as described earlier (Fendrich *et al*, 2007): negative=less than 5% of tumour cells positive; +=<50% of tumour cells positive; ++=>50% of tumour cells positive.

To prevent a selection bias we calculated survival curves for patients with a follow up of more and less than 12 months. The follow-up period of each patient is listed in Table 1. Ten functioning, small adenomas from patients with either Cushing's or Conn syndrome (see Table 2) served as benign control tumours.

The study protocol met the guidelines of the local ethics committee.

### Surgery and diagnostic work-up

We scheduled patients for surgery if a large tumour suspicious of ACC with or without hormone excess was found. In case of distant metastases, patients underwent surgery only to control the hormone excess. The standard diagnostic work-up is comprised of thin collimation CT of lung and abdomen. A careful hormonal assessment was obligate to exclude pheochromocytoma, subclinical Cushing syndrome and to identify potential tumour markers.

We used a thoracoabdominal incision and performed an *en bloc* resection including the adjacent periadrenal fat and lymph nodes between the diaphragm, the renal vein and the vena cava, or the aorta, respectively.

### Immunohistochemistry

For immunolabelling, formalin-fixed and paraffin-embedded archived tumour samples and corresponding normal tissues were stained as described earlier (Esni *et al*, 2004). Briefly, slides from archived ACCs were heated to 60°C for 1 h, deparaffinised using xylene, and hydrated by a graded series of ethanol washes. Antigen retrieval was accomplished by microwave heating in 10 mM sodium citrate buffer of pH 6.0 for 10 min. For immunohistochemistry (IHC), endogenous peroxidase activity was quenched by 10 min incubation in 3% H_2_O_2_. Non-specific binding was blocked with 10% bovine serum. Sections were then probed with a primary antibody against Snail (Santa Cruz, Santa Cruz, CA, USA) with a working dilution of 1 : 100, a primary antibody against Ki-67 (Dako, Denmark) with a working solution of 1 : 200 or a primary antibody for E-cadherin (Zymed, SF, CA, USA) with a working solution of 1 : 200, overnight at 4°C. The next day, bound antibodies were detected using the avidin–biotin complex (ABC) peroxidase method (ABC Elite Kit, Vector Labs, Burlingame, CA, USA). Final staining was developed with the Sigma FAST DAB peroxidase substrate kit (Sigma, Deisenhofen, Germany).

Mammary carcinoma samples from our tissue bank, that had previously been shown to express high levels of Snail, were used as positive controls with each batch of Snail IHC staining.

### RNA extraction and real-time quantitative PCR

A portion of available fresh frozen tumour tissue was homogenised and lysed with 600 *μ*l buffer RLT and whole RNA was extracted using the RNeasy kit (Qiagen, Hilden, Germany) with on-column DNA digestion following the standard protocol provided by the manufacturer. The mRNA was reverse transcribed into cDNA with oligo-dT primers using the Superscript 1st Strand System for RT–PCR (Invitrogen, Carlsbad, CA, USA) at 42°C for 50 min. All PCRs were carried out on a 7500 Real-Time PCR System (Applied Biosystems, Foster City, CA, USA) over 40 cycles, with denaturation for 15 s at 95°C and combined annealing/extension at 60°C for 1 min. Following an activation step at 95°C for 10 min, determination of Snail mRNA expression was performed over 40 cycles with 15 s of denaturation at 95°C and annealing/extension/data acquisition at 60°C for 60 s using the Power SYBR® Green PCR kit (Applied Biosystems).

Primer sequences for Snail were forward: 5′-tcccgggcaatttaacaatg-3′ and reverse: 5′-tgggagacacatcggtcaga-3′. Relative fold mRNA expression levels were determined using the 2^−Δ Δ *C*_*t*_^ method (Livak KJ *et al*, 2001) with ribosomal protein, large, P0 (*RPLPO*) as housekeeping control. *RPLP0* primer sequences were forward 5′-tgggcaagaacaccatgatg-3′ and reverse 5′-agtttctccagagctgggttgt-3′.

### Statistical analysis

Survival curves were computed from the time of surgical exploration to either death or most recent contact by using the Kaplan–Meier method. Log-rank test was applied to identify significant differences. For analysing proportions a two-tailed Fisher's exact test was used. *P*-values <0.05 were considered as statistically significant. Data were analysed using SPSS version 14.0 for Microsoft Windows.

## Results

### Patients

Twenty-six patients (8 male and 18 female) with ACC were included in this study. The median age of diagnosis was 47.5 years (range, 15–83 years). Patients were followed for a median period of 16 months (range, 5–153 months). Out of 26 patients one was diagnosed at stage I, 13 at stage II, six at stage III and six at stage IV according to the TNM classification. Six patients presented with distant metastasis at initial diagnosis, another nine patients developed them during further follow-up. Clinical and histological data are shown in Table 1.

Clinical data on 10 functioning adenomas, which served as benign control tumours, is summarised by Table 2.

### Expression of Snail

**Figure 1:**
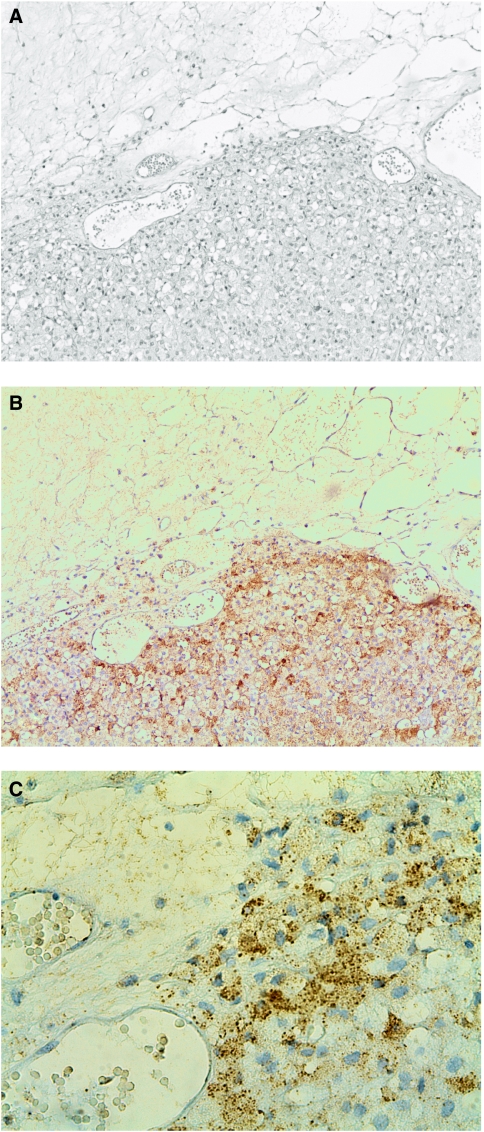
HE staining and IHC staining for Snail and E-cadherin in a human ACC ( × 10). (**A**) HE staining with tumour invading the surrounding fatty tissue. (**B**) Consecutive section with IHC staining for Snail showing Snail-expressing cells invading the fatty tissue. (**C**) Same specimen demonstrating the invasive front at a higher magnification ( × 40).

Overall, 17 of 26 (65%) ACC samples revealed positive immunohistochemical staining for Snail. Snail expression was inhomogeneously distributed in all positive tumour samples, revealing focal high expression close to the tumour's capsule in 8 out of 17 ACCs (Figure 1A and B). Seven of 8 of these tumours revealed a strong expression (++) of Snail at the invasive front. As seen in Figure 1B nearly every single cell along the capsular invasion showed a positive staining for Snail.

Interestingly, 2 out of 3 metastases (one LNM, one liver metastasis) studied showed a strong expression (++) of Snail.

**Figure 2:**
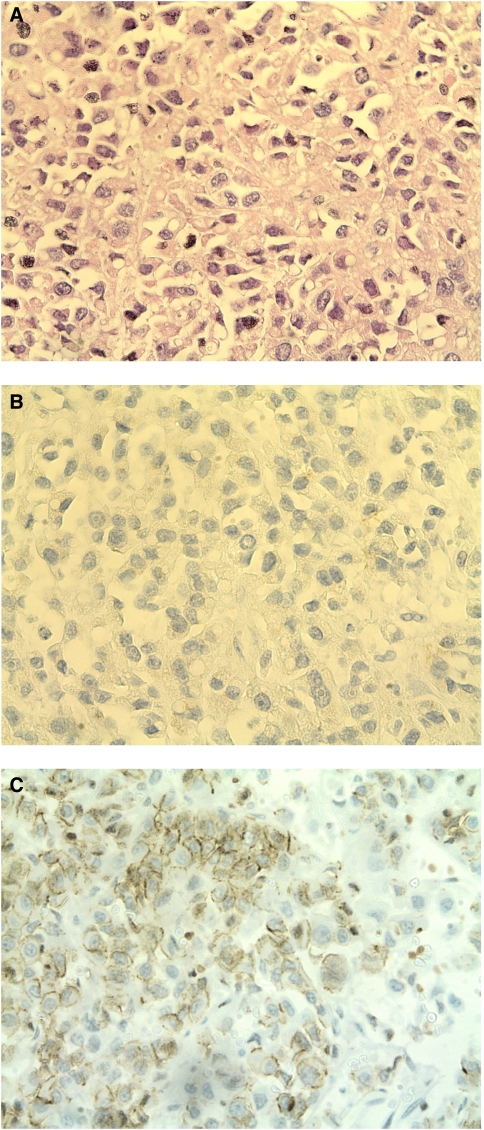
HE staining and IHC staining for Snail and E-cadherin in a Snail-negative ACC. (**A**) HE staining showing irregular shaped cells with mitoses. (**B**) Cells bared any Snail expression in the IHC. (**C**) Consecutive section of (**A**) and (**B**) revealing E-caherin expression in some cells indicated by the dark line associated to the cellular membrane.

Nine of 26 ACCs and one LNM were Snail negative (Figure 2B). In normal adrenocortical tissue, Snail expression could not be detected by immunohistochemistry apart from a population of single cells closed to the adrenal capsule. As expected, adjacent normal mesenchymal tissue revealed Snail expression and was used as internal positive control.

These results could be confirmed at the mRNA level on a subset of 10 samples using quantitative real-time RT–PCR. Normal adrenocortical tissue and Snail-negative tumours revealed lower Snail expression levels than + and ++ classified tumour samples (Figure 4), *P*=0.42. The difference between + and ++ tumours was not statistically different.

### Snail expression and stage of disease

Snail expression was associated with advanced stage of disease. Eleven of 12 patients with stage III and IV were Snail positive whereas only 6 of 14 patients with stage I and II revealed Snail expression (*P*=0.01). Five of six patients presented with synchronous distant metastases revealed Snail expression in the primary tumour, whereas only 12 out of 20 patients without metastases revealed Snail expression without reaching statistical significance (*P*=0.37). On account of the limited number of cases Snail expression and tumour stage did not show statistical significance for all stages if analysed separately.

### Snail expression, survival and developing of metastases

Survival rates were decreased in Snail-positive tumours in comparison to Snail-negative tumours: 10 out of 16 *vs* one out of eight patients succumbed to the disease after a median follow up of 14.5 (5–132) and 28.5 (6–153) months, respectively (*P*=0.03). In each group of Snail-negative and -positive tumours one unrelated death occurred and patients were therefore excluded. Survival curves were calculated including patients with a follow-up of more and less than 12 months (Figure 5A and B). Indeed both analyses revealed statistical significance (*P*=0.02 and *P*=0.01). The median actuarial survival was 127 months and 34 months for snail-negative and snail-positive tumours, respectively. The 2- and 5-year survival rate for Snail-negative and Snail-positive tumours were 100 *vs* 50%, and 80 *vs* 20%, respectively.

Patients with Snail-expressing tumours tend to develop distant metastases more frequently in the subsequent follow up: 7 of 11 patients developed distant metastases if Snail was expressed by the tumour, whereas only two of eight patients when Snail expression of the primary tumour was lacking (*P*=0.19).

If Snail-positive tumours classified as + and ++ were analysed separately differences, risk of distant metastases, survival and stage of disease were statistically not significant.

### Ki-67 and Snail

Interestingly, we observed the highest median Ki-67 index in strongly Snail (++) expressing ACCs with 10% (range, 1–30) of cells staining positive for Ki-67. The median Ki-67 index decreases in Snail+ tumours with 5% (range, 1–10) to reach 2.5% (range, 1–70) in Snail-negative tumours. The differences failed to reach statistical significance.

### E-cadherin expression

Twenty-three of 26 (88%) ACCs did not show any E-cadherin expression. In three tumour samples, we detect E-cadherin and it was remarkable that these three patients were without recurrence after a follow-up of 6, 40, and 50 months, respectively.

### Snail and E-cadherin expression in adrenocortical adenomas

**Figure 3:**
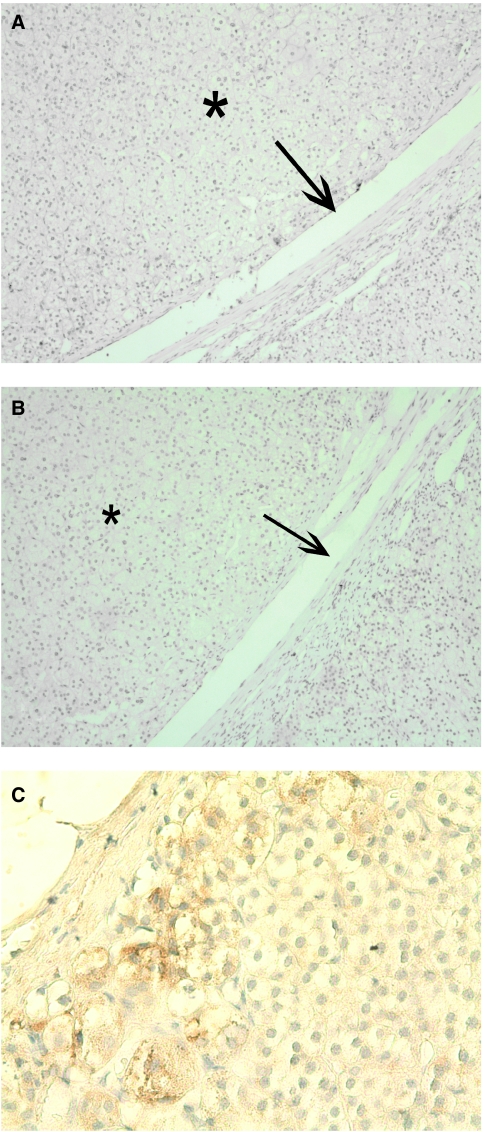
IHC for Snail and E-cadherin in an adrenocortical adenoma and Snail expression in normal adrenal cortex. (**A**) Adrenal cortical adenoma showed no Snail expression. Arrow indicates the border between normal adrenal cortex and adenoma (^*^). (**B**) Nor normal adrenocortical tissue neither adenoma showed any E-cadherin expression in this consecutive section. (**C**) Normal adrenocortical tissue was predominantly negative for Snail, but some single Snail-positive cells within the zona glomerulosa closed to the capsule and some Snail-positive mesenchymal cells (not shown) could be detected.

We analysed E-cadherin and Snail expression in 10 adrenocortical adenomas. Clinical data are shown in Table 2. None of these 10 functioning adenomas (five patients with Cushing and five patients with Conn syndrome) revealed Snail expression by IHC, and in only 3 out of 10 we detected E-cadherin expression (see Figure 3A and B).

## Discussion

Local tumour invasion is an early and essential step in the metastatic cascade. In epithelial tumours, this is often closely associated with the acquisition of mesenchymal cell-like characteristics; a process termed EMT. One of the hallmarks of EMT is the functional loss of E-cadherin, which is known to promote cell-to-cell adhesion and consecutively gives rise to invasion and progression. The zinc-finger transcription factor Snail is a potent repressor of E-cadherin and induces EMT. In ovarian, colon, breast and hepatocarcinoma Snail overexpression has been observed and resulted in E-cadherin loss. In hepatocarcinoma and breast cancer, increased Snail expression was associated with distant metastases and poorer clinical outcome (Miyoshi *et al*, 2005; Come *et al*, 2006). In colon and ovarian carcinoma patients with Snail-expressing tumours, experienced a higher risk for distant metastases (Elloul *et al*, 2005; Roy *et al*, 2005). To the best of our knowledge, this is the first study of Snail expression in ACCs and adrenocortical tumours in general.

**Figure 4:**
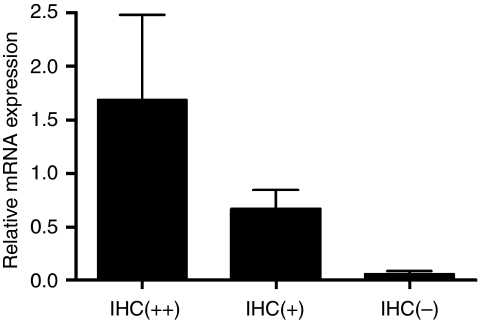
Steady state Snail mRNA levels were determined by real-time RT–PCR in a subset of 10 samples.

We found that Snail is expressed in about two-third of a large series of ACCs. Snail-expressing cells were mostly found at the invasive front of the ACCs as seen in tumours induced in the skin of mice (Cano *et al*, 2000) and recently described by our group in neuroendocrine tumours of the ileum (Fendrich *et al*, 2007). At this site, tumour cells migrate into and invade the surrounding tissue either as single cells or in collective clusters and give evidence of an EMT (Zhou *et al*, 2004). Snail-positive invasive fronts were observed in 8 of 17 Snail-positive ACCs (Figure 1). In normal adrenocortical tissue, Snail expression was mostly absent apart from a distinct cell population close to the capsule (Figure 3C). We confirmed Snail expression levels by RT–PCR with cDNA derived from whole tumour samples (Figure 4). Expression levels correlated with data established by IHC although a discrimination of specific tumour regions as invasive areas could only have been achieved by microdissection.

Remarkably and most importantly, we found that Snail expression strongly correlates with clinical outcome.

First, Snail expression was associated with an increased risk for distant metastases. Once a diagnosis of cancer is established, the urgent and fearful question is whether it is localised or has already spread to regional lymph nodes and visceral organs. Despite all improvements in diagnosis, surgery and adjuvant therapies, most deaths of patients with cancer result from the growth of metastases. This is also true for ACCs. In this study, seven of 12 patients with Snail-expressing tumours without DM at the initial diagnosis developed distant metastases, whereas only two of eight patients developed distant metastases when Snail expression of the primary tumour was lacking. This is in line with recent reported studies for other tumour entities, such as breast (Zhou *et al*, 2004), ovarian (Elloul *et al*, 2005), colon (Roy *et al*, 2005), and squamous cell carcinoma (Takeno *et al*, 2004). The first of the many steps leading to metastasis – the acquisition of local invasiveness involves major changes in the phenotype of cancer cells within the primary tumour. To acquire motility and invasiveness, carcinoma cells must shed many of their epithelial characteristics, detach from epithelial sheets, and undergo a drastic phenotypic alteration. Therefore, the expression of Snail represents a specific stage of tumour progression, preparing tumour cells to invade and to metastasise, reflected by our findings. In addition, two of three resected distant metastases expressed Snail, which strongly supports this hypothesis. Studies in breast carcinoma have shown similar results (Moody *et al*, 2005). Recently, it has been proposed that Snail-mediated tumour recurrence is associated with self-renewal in cancer stem cell-like cells (De Craene and Berx, 2006). In this regard, snail expression of single cells in the zona glomerulosa of normal adrenal cortex close to the organ capsule (see Figure 3C) may indicate an adrenal stem cell, so called adrenocyte, which has been proposed by several groups (Skelton, 1959; Belloni *et al*, 1990; Feige *et al*, 1998). Thus, our study might also indicate a role of Snail expression as a prognostic marker in ACC. Patients with Snail-positive ACCs, but without distant metastases at initial operation should undergo a very close follow up, because they might be at a higher risk to develop distant metastases.

Second, patients with Snail-expressing ACCs were diagnosed more often in advanced disease, which also underlines that expression of Snail, could be seen as one of the critical steps in ACC progression. Eleven of 12 patients with stage III and IV were Snail positive whereas only 6 of 14 patients with stage I and II revealed Snail expression (*P*=0.01). Because, virtually all patients with ACC die due to distant metastases, the appearance of Snail expression probably occurs shortly before invasion and start of metastasis.

**Figure 5:**
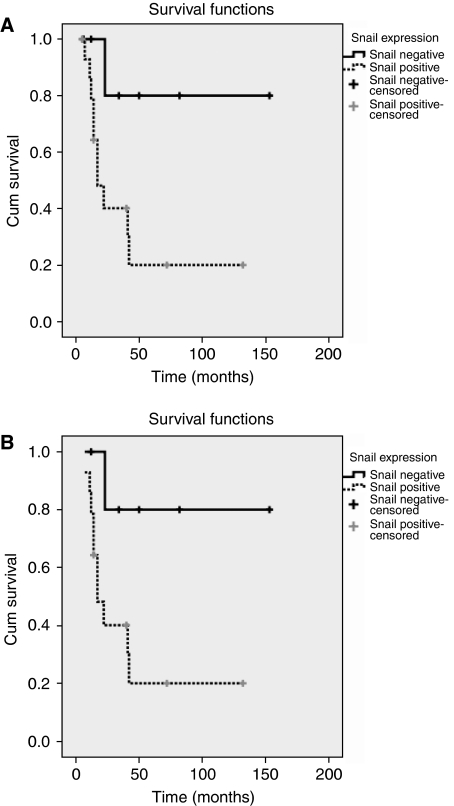
Kaplan–Meier curve of patients with positive and negative Snail expression: Note the decreased survival of Snail-expressing ACCs. (**A**) Survival in 24 patients with ACC (two were excluded because of disease-unrelated death), *P*=0.02. (**B**) Survival in 20 patients with ACC (four were excluded as the follow-up was minor 12 months, two were excluded due to disease-unrelated death), *P*=0.01.

Third, patients with Snail-expressing ACCs had a decreased survival rate (Figure 5A). Survival rates were significantly lower in Snail-positive tumours than in Snail-negative tumours (*P*=0.02). In addition, we analysed survival rates for patients with follow-up longer than 1 year to avoid bias because of missed metastases during shorter follow up (Figure 5B) and had very similar median follow up of 14.5 (snail positive) and 28.5 (snail negative) months in the groups. A decreased survival for patients with Snail-expressing tumours is in line with recent studies. In ovarian cancer, a high Snail mRNA expression predicted a shorter effusion-free survival in ovarian cancer (Elloul *et al*, 2005). In node-negative invasive ductal breast carcinomas, Snail mRNA expression correlated with disease-free and overall survival (Toyama *et al*, 2006). The decreased survival in our study might be either a consequence of the more advanced tumour stage and the presence of distant metastases in patients with Snail-positive tumours, or it might reflect an additional aggressiveness of the tumour cells.

Only 3 of 26 ACCs revealed E-cadherin expression by immunohistochemistry. Interestingly all three patients were alive without evidence of metastases or recurrence. The lack of E-cadherin expression is in line with the overexpression of snail and indicates EMT in ACC.

Histopathological scoring of adrenocortical tumours provide a tool for defining malignancy, survival and the risk of metastases as shown by van't Sant *et al* (2007). However, some tumours do not match all criteria enclosed in the scoring system and therefore do not reach the threshold for malignancy. Despite these facts two patients with adrenocortical tumours classified as benign in the former study developed distant metastases. We enroled three patients with W and VS of 2 out of 5.7, 3 out of 8 and 2 out of 5.7, respectively. All patients presented with large (130, 75 and 70 mm) and hormonally active tumours (one Conn Syndrome, two testosteron and cortisol-producing tumours). They displayed high Ki-67 indexes of 10, 3 and 30%, respectively. A Ki 67 index of 2.5–5% is suggestible for an ACC (Wachenfeld *et al*, 2001; Sasano *et al*, 2006). Indeed scoring indices could reliably discriminate between malignant and benign tumours as proven by several studies (van Slooten *et al*, 1985; Weiss *et al*, 1989).

In conclusion, we found that Snail is expressed in a high proportion of ACCs, which is in line with the concept of EMT. Furthermore, we show that Snail expression is associated with decreased survival, advanced disease and tendency of an enhanced risk for distant metastases, suggesting that the Snail expression could be used as a prognostic marker in ACC.

## Figures and Tables

**Table 1:** Clinical characteristics, results of Snail and E-cadherin immunohistochemistry in 26 patients with ACC

**Patient no.**	**Age**	**Sex**	**Histo**	**Size/Site**	**TNM**	**Ki-67 (%)**	**Scoring (W/VS/H)**	**E-cad IHC**	**Snail IHC**	**Follow up (months)**	**Distant metastases**
1	57	F	ACC	200/l	III	10	4/9/3.11	−	++	NED (132)	N
2	43	F	ACC	170/r	IV	10	6/20/4.52	−	++	DOD (14)	Y
3	42	M	ACC	170/l	III	10	5/18/3.13	−	++	DOD (41)	Y
4	53	F	ACC	100/l	III	20	4/23.5/1.68	−	++	DOD (17)	Y
5	73	F	ACC	85/l	III	1	5/12.3/1.89	−	++	DURC (15)	N
6	39	F	ACC	130/l	II	10	2/5.7/1.69	−	++	NED (72)	N
7	37	F	ACC	90/l	III	30	5/14.7/4.5	−	++	DOD (42)	Y
8	46	F	ACC	30/r	I	10	4/9/2.61	+	++	NED (40)	N
9	49	F	ACC	70/l	II	2	5/13.7/2	−	++	NED (14)	N
10	72	F	ACC	60/l	IV	5	7/17.8/3.29	−	++	DOD (14)	Y
11	26	M	ACC	80/r	IV	20	ND	−	++	DOD (12)	Y
12	34	F	ACC	120/r	III	1	4/14.7/2.29	−	+	DOD (22)	Y
13	46	M	ACC	105/r	IV	10	6/24.2/3.92	−	+	DOD (7)	Y
14	50	F	ACC	95/r	II	5	5/9//4.13	−	+	DOD (17)	Y
15	41	M	ACC	210/l	II	1	7/17.8/3.89	−	+	DOD (11)	Y
16	69	M	ACC	100/r	IV	30	7/20.1/3.52	−	+	AWD (5)	Y
17	72	M	ACC	95/l	II	5	4/10.4/2.6	−	+	NED (5)	N
18	24	F	ACC	75/r	II	3	3/8/1.31	+	−	NED (6)	N
19	22	F	ACC	120/r	II	1	5/21.1/2.83	−	−	DURC (15)	N
20	15	M	ACC	120/l	II	2	5/19.1/2.6	−	−	DOD (23)	Y
21	72	M	ACC	110/r	II	5	6/26.8/4.52	−	−	AWD (82)	Y
22	83	F	ACC	120/l	II	30	8/26.8/3.89	+	−	NED (50)	N
23	58	F	ACC	60/r	II	2	4/12.1/2..23	−	−	NED (34)	N
24	55	F	ACC	150/l	IV	20	8/26.8/5.21	−	−	AWD (12)	Y
25	50	F	ACC	100/l	II	2	4/8/1.68	−	−	R(108), NED(153)	N
26	43	F	ACC	70/l	II	20	2/5.7/1.69	−	−	NED (6)	N

ACC=Adrenocortical Carcinoma; AWD=Alive with disease; DURC=death of unrelated cause; DOD=Dead of disease; E-cad=E-cadherin expression; H=Hough; IHC=Immunohistochemistry; NED=No evidence for disease; VS=van Slooten; W=Weiss.

**Table 2:** Clinical characteristics, results of Snail and E-cadherin immunohistochemistry in 10 patients with benign adrenocortical adenomas

**Pat no.**	**Age**	**Sex**	**Syndrome**	**Size/side**	**E-cad**	**Snail**
1	45	F	Cush	22/l	−	−
2	33	M	Cush	25/l	−	−
3	24	F	Cush	30/r	+	−
4	36	F	Cush	20/l	+	−
5	46	F	Cush	15/l	−	−
6	64	F	Conn	20/l	−	−
7	49	F	Conn	21/r	−	−
8	64	M	Conn	15/l	+	−
9	38	F	Conn	15/l	−	−
10	69	M	Conn	21/r	−	−

Conn=Conn syndrome; Cush=Cushing's syndrome; E-cad=E-cadherin expression; F=female; l=left; r=right; M=male.
